# Supplementary material for: Pheomelanin pigment remnants mapped in fossils of an extinct mammal
Source: Nat Commun. 2019 May 21;10:2250. doi: 10.1038/s41467-019-10087-2 (PMC6529433; doi:10.1038/s41467-019-10087-2)
Supplement: Supplementary file 1 — Supplementary Information [file 41467_2019_10087_MOESM1_ESM.pdf]

Supplementary Information

Pheomelanin pigment remnants mapped in fossils of an extinct mammal

Authors:

Phillip L. Manning[1,2], Nicholas P. Edwards[3], Uwe Bergmann[4], Jennifer Anné[5], William I. Sellers[1], Arjen van Veelen[6], Dimosthenis Sokaras[4], Victoria M. Egerton[1], Roberto Alonso-Mori[7], Konstantin Ignatyev[8], Bart van Dongen[1], Kazumasa Wakamatsu[9], Shosuke Ito[9], Fabien Knoll[10], and Roy A. Wogelius[11,]*.

Affiliations:

[1]University of Manchester, School of Earth and Environmental Sciences, Interdisciplinary Centre for Ancient Life, Manchester, M13 9PL, UK
[2]Department of Geology and Environmental Geoscience, College of Charleston, 66 George St, Charleston, SC 29424, USA
[4]Stanford PULSE Institute, SLAC National Accelerator Laboratory, Menlo Park, CA, 94025, USA
[3]Stanford Synchrotron Radiation Lightsource, SLAC National Accelerator Laboratory, Menlo Park, CA 94025, USA
[5]Children's Museum of Indianpolis, P.O. Box 3000, Indianapolis, IN 46206-3000, USA
[6]University of Southampton, Engineering and the Environment, Highfield, Southampton SO17 1BJ, UK
[7]Linac Coherent Light Source, SLAC National Accelerator Laboratory, Menlo Park, CA 94025, USA
[8]Diamond Light Source, Didcot, OX11 0DE, UK
[9]Department of Chemistry, Fujita Health University School of Health Sciences, Toyoake, Aichi, Japan
[10]ARAID—Fundación Conjunto Paleontológico de Teruel-Dinópolis, 44002 Teruel, Spain
[11]University of Manchester, School of Earth and Environmental Sciences, Williamson Research Centre for Molecular Environmental Science & Interdisciplinary Centre for Ancient Life, Manchester, M13 9PL, UK

*corresponding author: R.A.W. (email: roy.wogelius@manchester.ac.uk)

Supplementary Note 1 : Melanin chemistry- background

The physical properties of a melanin are a function of what they are bound to (e.g. proteins, metal ions.). These heterogamous polyphenol-like biopolymers vary in colour from yellow to black[1]. Melanins are not directly derived from diet, unlike carotenoids, but are manufactured endogenously. The enzyme tyrosinase is central to melanogenesis in melanosomes in all vertebrates[1]. Copper is the metal co-factor for tyrosinase. There is variation in size between the two main melanin molecules, with eumelanin forming larger, rod-like granules that are insoluble in almost all solvents. Pheomelanins, in contrast, form the reddish-brown pigments. They are smaller, globular granules compared to eumelanin and are soluble in alkaline solutions[2]. Pheomelanins also have different light absorbance and structural characteristics to eumelanins. Ito[3] suggested that eumelanin is deposited on preformed pheomelanin and that the ratio of the two was determined by tyrosinase activity and cysteine concentration, but recently it has been shown that this ratio is a function of tyrosinase activity and the availability of tyrosine and cysteine in melanosomes[4,5]. A number of transition metal ions (e.g. Cu^{2+}, Co^{2+} and Zn^{2+}) with a wide distribution in biological systems clearly affect the chemical properties of melanin formed by the action of tyrosinase[5,6].

Melanin pigments consist of several covalently linked indoles and are considered unusually large polymers compared to most other natural pigments[7]. All melanin-containing tissues contain both eumelanin and pheomelanin. Total melanin concentration is less significant in shaping colour variability than is the relative proportion of the two pigment types. Melanin can also occur in tissue where other colour pigments (e.g. carotenoids) are present. Melanin granules, however, differ in size, shape and

colour between different species[8,9]. Whereas eumelanin is insoluble in almost all solvents and resistant to chemical treatment, pheomelanin is soluble in dilute alkali[10].

Supplementary Note 2: Geological setting

The Willershausen conservation lagerstätte is a Pliocene (Placenzian, ~3 million years old) lacustrine deposit that was laid down in a sinkhole formed due to the dissolution of underlying Permian aged evaporites located in the Harz Mountains, Germany. Taphonomic conditions at the Willershausen site gave rise to exceptional preservation of plants and arthropod cuticles, but also to rare vertebrate remains[11]. Remarkable fossils of the rodent *Apodemus atavus* discovered at Willershausen clearly show rare soft tissue preservation, including fur, skin and even stomach contents[12]. Thus, the well-preserved rodents from Willershausen provide a rare opportunity for studying the fossil remains of mammalian soft tissue.

The depositional basin was small, no more than 10 metres deep and between 150-200 metres in diameter[11,13]. Light coloured laminated marls were deposited near shore, whilst darker finely laminated marls typify offshore anoxic facies[14]. This freshwater lake was subject to increases in salinity, influenced by underlying (Zechstein) evaporite deposits[14,15], thus explaining the abundant presence of halophile diatoms[16,17]. Ferguson & Knobloch[18] suggest the presence of brine-caused stratification of the Willershausen lake waters, with reduced turnover leading to the bottom waters becoming heavier than those at the surface. The static water column led to anoxic conditions in the lake bottom. Furthermore, dolomitization reactions indicate that this was an alkaline system[13]. The exceptional preservation of organic material also extends to the significant survival of chitin (40%) from the anoxic portion of the lake[16,19,20].

Supplementary Note 3: Details of sulfur oxidation

A small amount of benzothiazole (3%) was resolved for the bone spectrum, which indicates that there may be traces of organic S present within or coating these bones. Interestingly, the graminoid (grass) fragment shows no organic ring coordinated sulfur, but does show trace cysteine, disulphide, and sulfoxide species along with a strong sulfate signal.

The pertinent reactions are:

(1) *Cystine* + *benzothiazole* + water -> 2 <u>cysteine</u> + <u>methionine sulfoxide</u>

[>C-S-S-C<] + [>C-S-C<] + H_2O -> 2 [>C-S-H] + [>C-(S=O)-C<]

(2) 2 <u>methionine sulfoxide</u> + oxygen -> carbon dioxide + <u>sulfate</u>

2[>C-(S=O)-C<] + 7 O_2 -> 4 CO_2 + 2 SO_4^{-2}

Compounds in italics are reactants present in the pristine fur, while those that are underlined are products that are identified in the fossil. After the reaction has gone to completion only inorganic sulfate would remain.

The first reaction above represents the hydrolysis of keratin and pheomelanin and the second is the final oxidation reaction to create inorganic sulfate. The breakdown products cysteine, methionine sulfoxide, and sulfate are all visible in the spectrum taken from the fossil fur (Figure 3, Supplementary Table 1), where the bulk is heterocyclic sulfur and sulfate.

Conservative behaviour of sulfur in this system allows us to place a rough bracket on the time period we would expect the organic sulfur compounds to be preserved for in this specific geochemical environment. Assuming a linear reaction rate based on the loss of disulphide from the fur over time, we calculate an oxidation rate for organic sulfur of 8×10^{-21} mol g^{-1} s^{-1}. This is orders of magnitude slower

than the dissolution rate of quartz in near neutral pH solutions, and is consistent with the wide body of literature detailing the survival of organosulfur compounds in crude oils over hundreds of millions of years. In fact, the breakdown rate here is probably faster than in other environments, and predicts that after only 66 m.y. the organic sulfur in these fossils would be too dilute to resolve. We acknowledge that this is only an estimated oxidation rate and that the rate law will almost certainly not be a linear zero order equation, but in any case these calculations are geochemically reasonable and we may therefore use the progress of the reaction as analysed to, at minimum, place a constraint on possible reaction rates and thus give us a kinetic basis upon which to begin comparing the sulfur inventories of other exceptionally preserved fossils from different geochemical systems.

We finally note that because pheomelanin is soluble in alkali solutions, in order for pheomelanin residue to be preserved in situ at Willershausen, the sedimentation rate was probably high (at least episodically) in order to protect the soft tissue from solvent attack and enable the observed exceptional preservation to occur.

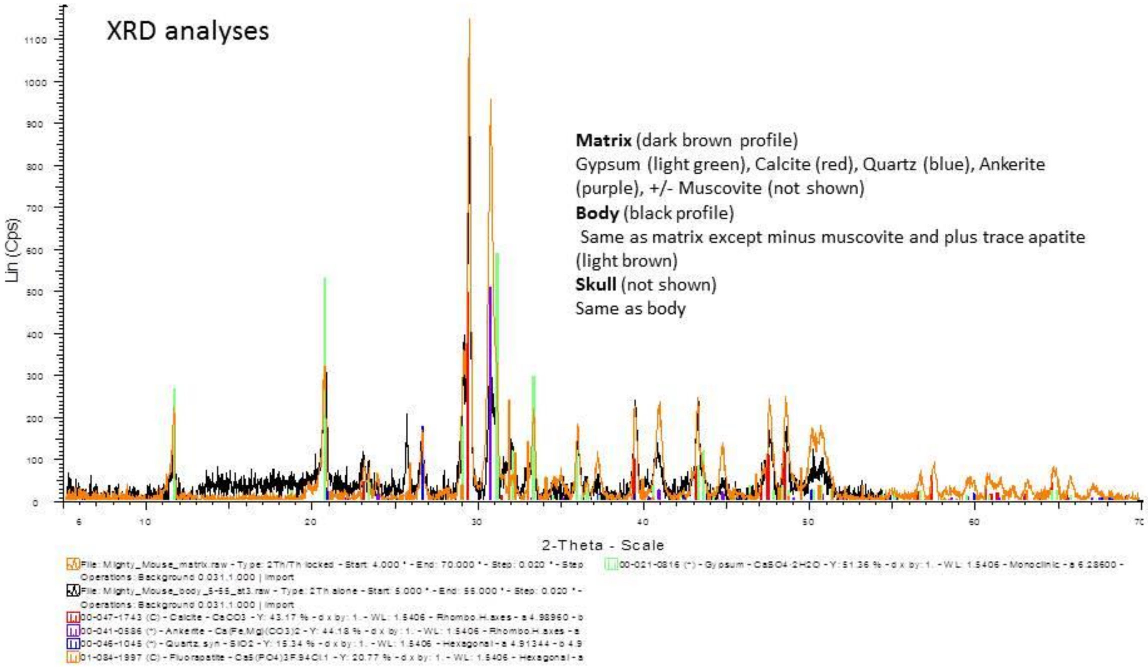

Supplementary Figure 1. **XRD analysis of the lateral fossil.** Sedimentary matrix (brown profile) compared to the body of the fossil (black profile).

Correlation maps of Zn and thiol for specimens 20027B (top) and 17393 (bottom). (Light colour indicates positive correlation. Target and source arrays were background subtracted in both cases).

Incomplete preservation and specimen position do not allow us to unequivocally resolve remnant patternation, however the correlation maps and related spectroscopy indicate with certainty that the dorsal surface of this species was phaeomelanin pigmented.

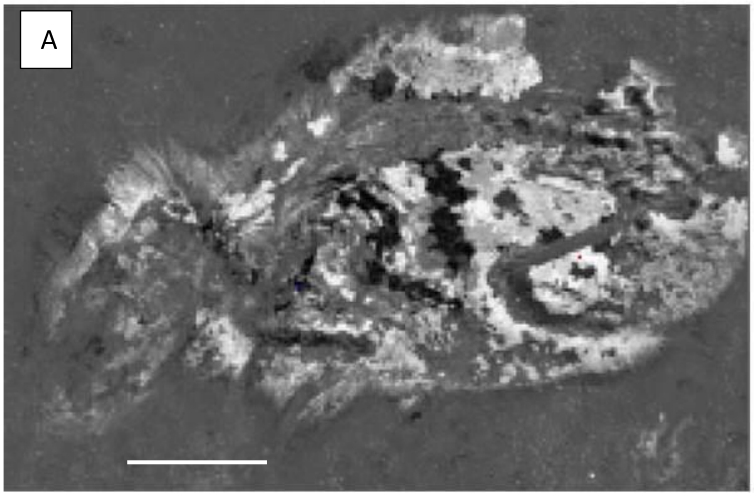

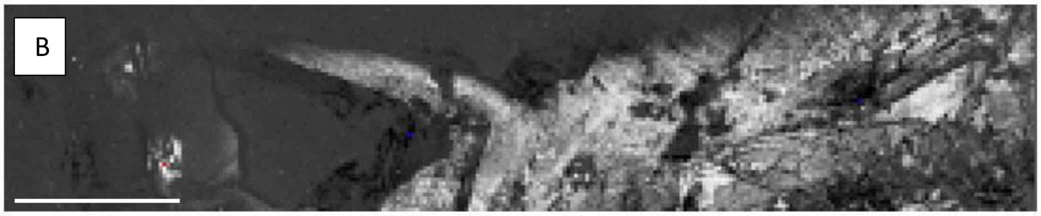

Supplementary Figure 2. **Correlation maps.** A) Zn correlation with organic sulfur in the dorsal fossil. B) Zn and organic sulfur in the lateral fossil. The R value calculated for Zn and organic sulphur over the entire surface shown in Supplementary Figure 2A is 0.64[21]. Correlation between Zn and organic S is not perfect for three main reasons, listed in order of importance: 1) the presence of non-sulphide minerals containing trace amounts of Zn in the sedimentary matrix (see the bright spots at lower right of Fig. 2A as well as diffuse low intensity background to lower left and upper right), 2) the presence of organic sulphur-bearing plant debris in the matrix which is low in Zn, and 3) technical difficulties in achieving perfect registration between the pixel coordinates of the low Z and high Z scans used to perform the comparison. Scale bars = 1 cm.

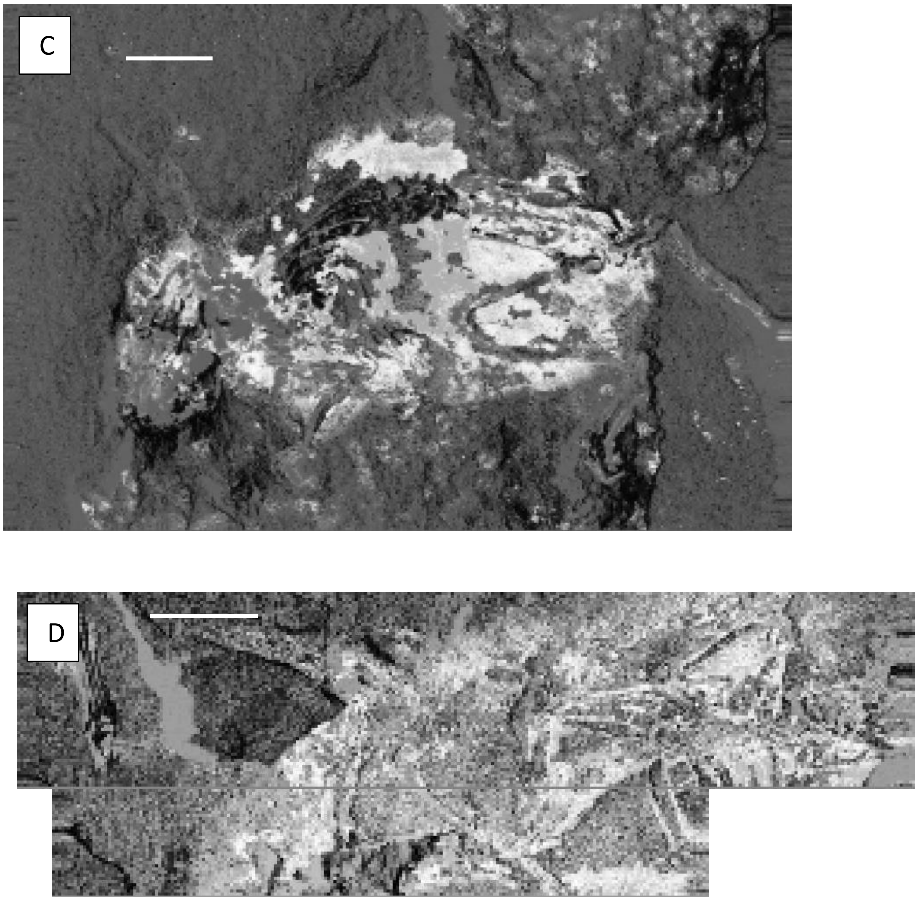

Supplementary Figure 2. **Correlation maps** (cont.). C) Zn correlated with Cu in the lateral fossil. D) Zn correlated with Cu in the dorsal fossil parts 1 and 2. Correlation produces similar results for all three scans, with an average R value of 0.73 (R^2 = 0.54). Cu and Zn are clearly correlated within the integument of the fossil, consistent with the presence of both eumelanin and pheomelanin derived residual chemistry. Scale bars = 1 cm.

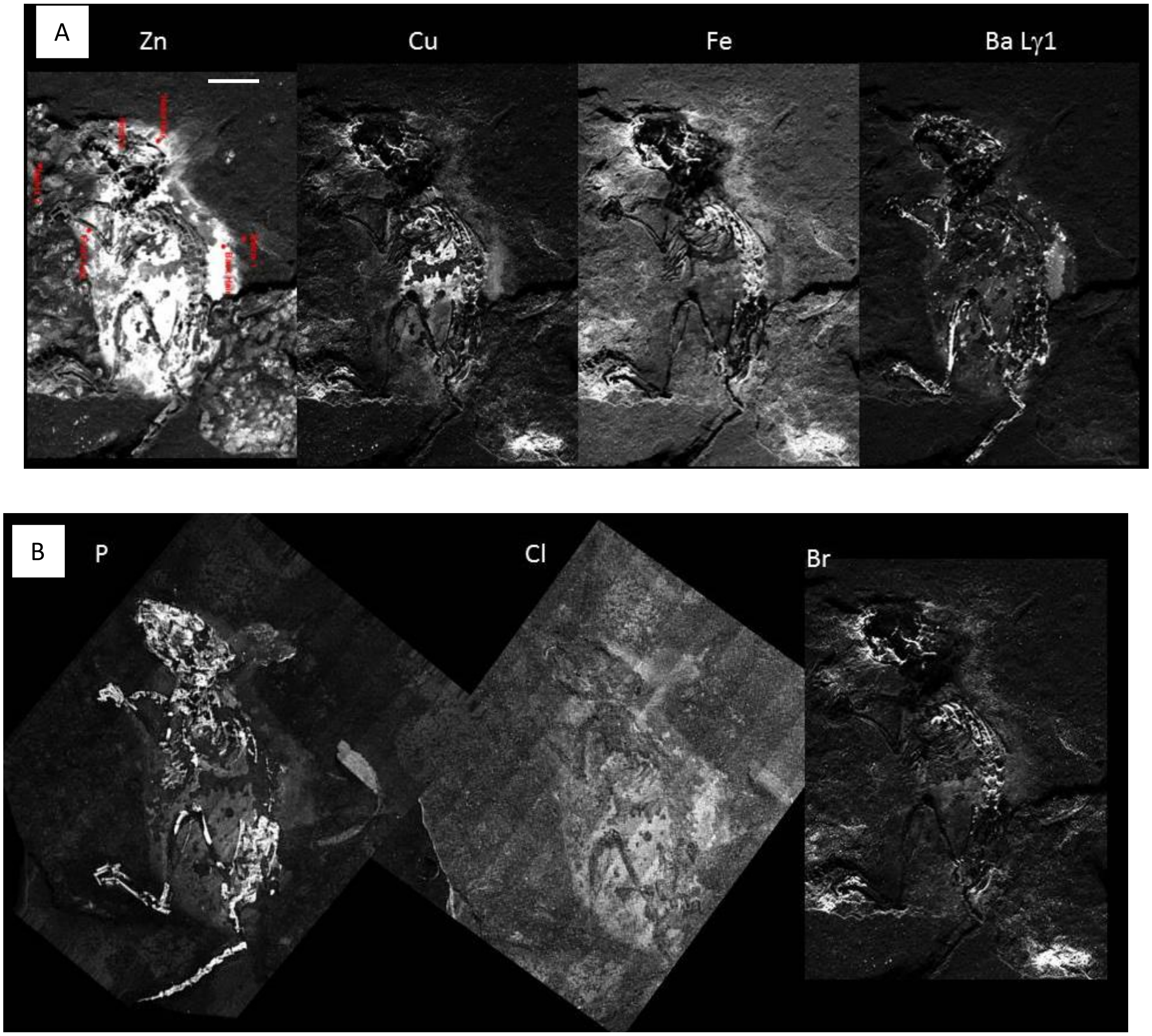

Supplementary Figure 3. **Individual SRS-XRF maps of selected elements in the lateral fossil.** The phosphorous distribution map not only allows us to clearly resolve the remnants of bone, but also shows that there is residual phosphorous associated with the integument (ears in particular) most likely derived from collagen residue. Scale bar = 1 cm.

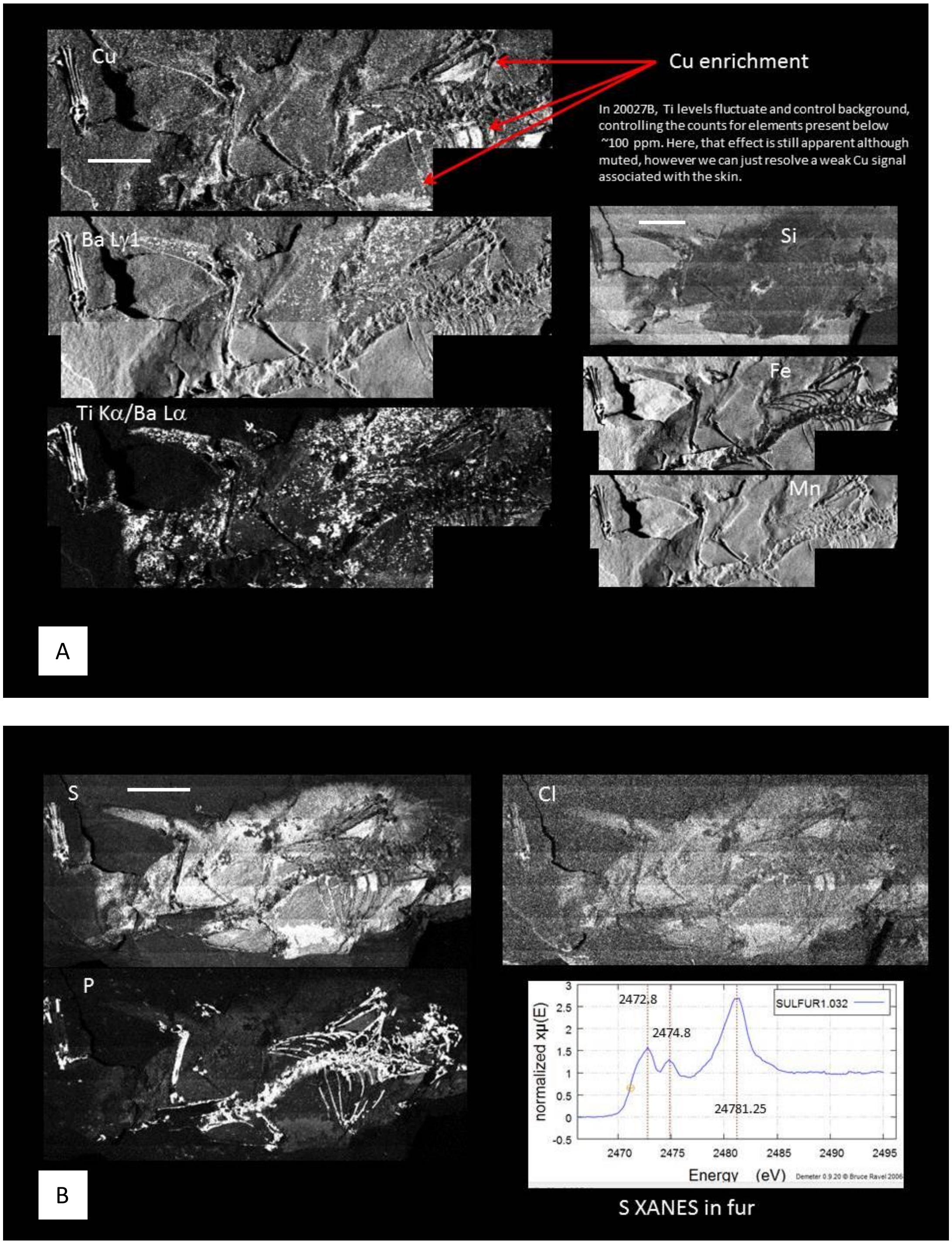

Supplementary Figure 4. **SRS-XRF maps of the dorsal fossil.** A. These highlight the distribution of Ti particulate. B. Light element individual maps of the dorsal fossil. Sulfate is present in the dorsal specimen on the bedding plane and as a "dusting" related to the periphery of the integument, which may be due to gypsum nucleating on the fur post mortem from the aqueous phase, or may represent complete oxidation of the precursor integument. Scale bars = 1 cm.

A

A. atavus fossil (DLS-22417)

N1 =O 3.111171
s02 0.801485 +/-0.039567
e0_1 2.817297
R1 1.986274
sig1 0.010161
N2=O 1.843113
R2 2.120008
sig2 0.001748
N3 =C 4.433129
R3 2.635143
sig3 0.005698
N4 =S 0.790403
R4 2.347785
sig4 0.002191
N5=Zn 5.438982
R5 3.843582
sig5 0.016515
N6=O(ms) 6.790102
R6 4.105746
sig6 0.041865
1 path red chi sq = 122.99
Df = 26, red chi crit = 1.55
2 paths, 100.69 pass
3 paths, 45.58, pass
4 paths, 32.44, pass
5 paths, 16.22 pass
6 paths, 14.61 pass/marginally
R-factor = 0.0603
Zn-Zn like a Histidine-proline glycoprotein

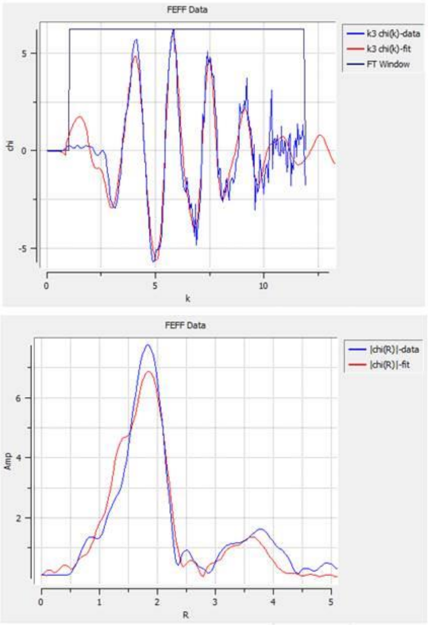

B

A. atavus fossil (DLS-3705)

N1=O 3.1477
s02 0.9
e0_1 0.662577
R1 2.012
sig1 0.0067
N2=O 0.270397
R2 2.137997
sig2 0.001
N3=C 1.784
R3 2.692743
sig3 0.0079
N4=S 0.50943
R4 2.320529
sig4 0.002443
1 path:red chi sq = 19.16
Df = 27, chi sq crit = 1.54
2 paths, red chi sq =16.51 (passes)
3 paths, red chi sq = 13.18 (passes)
4 paths, red chi sq = 8.22 (passes)
R factor = 0.0838
Approx. 14% S coordinated
Changing over to porphyrin model (next page) gives red chi sq = 5.95, passes test.

File: (A3705Mouse_merge.chik)

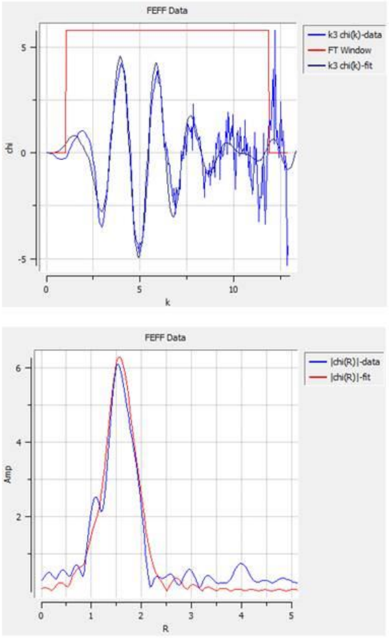

Supplementary Figure 5. **Fits to the EXAFS data.** A) and B) *A. atavus* lateral fossil.

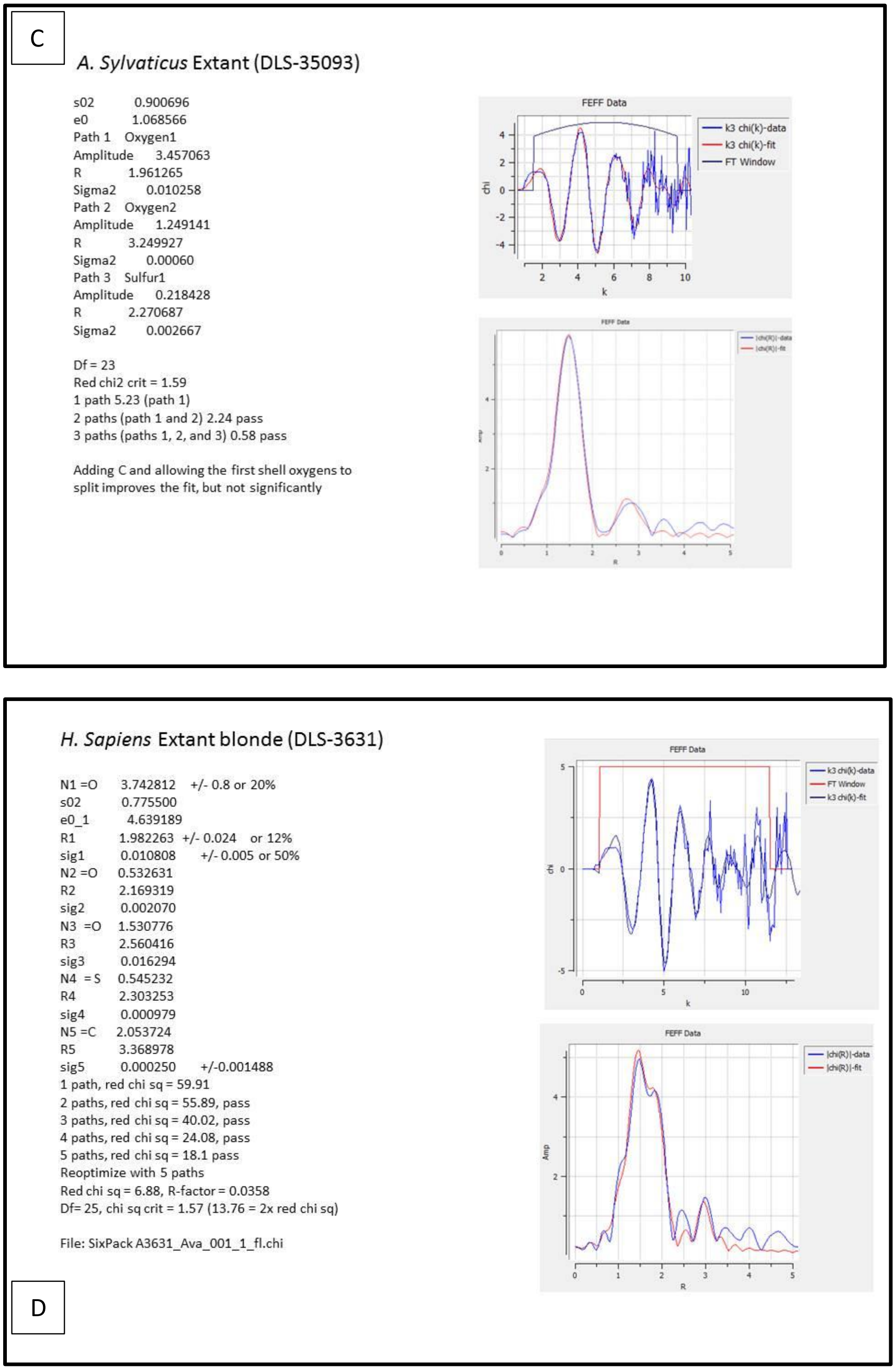

Supplementary Figure 5 (continued). **Fits to the EXAFS data**. C) *A. sylvaticus* extant. D) *H. sapiens* extant blonde.

E

Zn acetate standard (DLS-6051)

N1 =O 4.027877 (2)
s02 0.696641
e0_1 0.601317
R1 1.988843 (1.9871)
sig1 0.010193
N2 =O 0.330662 (4)
R2 2.051251 (2.1838)
sig2 0.001871
N3=C 0.918171 (2)
R3 2.688195 (2.5522)
sig3 0.001 (fixed)

(reference values in parentheses)

1 path, 24 df
del chi crit = 1.577
30.59 red chi sq

2 paths, 26.99 red chi q (passes)
3 paths, 19.6 red. Chi sq (passes)
R-factor= 0.0561

Additional O path fails

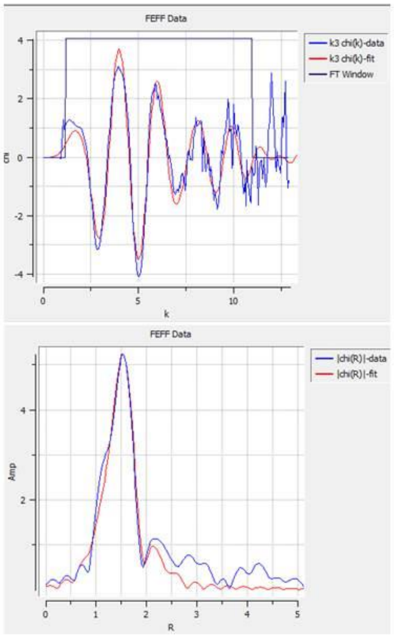

ZnSO4.7H2O Standard (DLS-6059)

All three shells are oxygen

N1 5.717111 (6)
s02 0.993767
e0_1 0.897166
R1 2.085242 (2.06-2.12)
sig1 0.009352
N2 4.326883 (4)
R2 4.151412 (4.1778)
sig2 0.014591
N3 0.718007 (24)
R3 3.266847 (3.5-3.7, water O double-scattering)
sig3 0.000904
in () are for ZnSO4.6H2O

Red chi sq =42.36 one path
24.50 two paths del_chi=17.86
21.55 three paths del_chi = 2.95
df = 17
del_chi (crit) = 1.34
R-factor = 0.0196

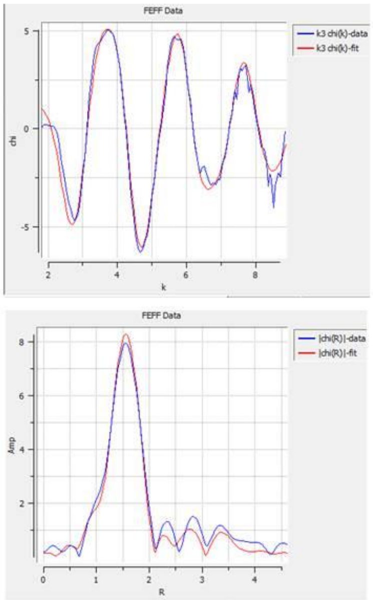

F

Supplementary Figure 5 (continued). **Fits to the EXAFS data**. E) Zn-acetate standard. F) ZnSO4 standard.

ZnS Wurtzite (DLS-6058)

N1 =S 4.345777 (4)
s02 0.9 +/-0.063042
e0_1 2.672918 (delEf = 2.321)
R1 2.330310 (2.3411)
sig1 0.008163
N2 =Zn 12.54729 +/-2.389475 (12)
R2 3.847676 (3.823)
sig2 0.017662
N3 =S 9.844612 +/-2.776050 (9)
R3 4.507560 (4.4834)
sig3 0.015598

ChiSq =141.01, RedChiSq = 8.05
18 df
Rfactor = 0.0534

Numbers in parentheses are theoretical values for wurtzite

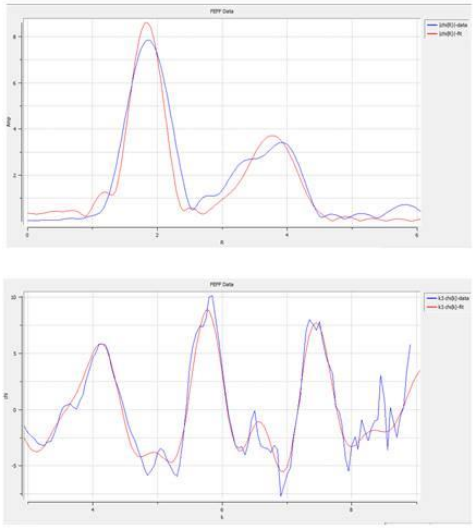

G

Supplementary Figure 5 (continued). **Fits to the EXAFS data**. G) ZnS (wurtzite).

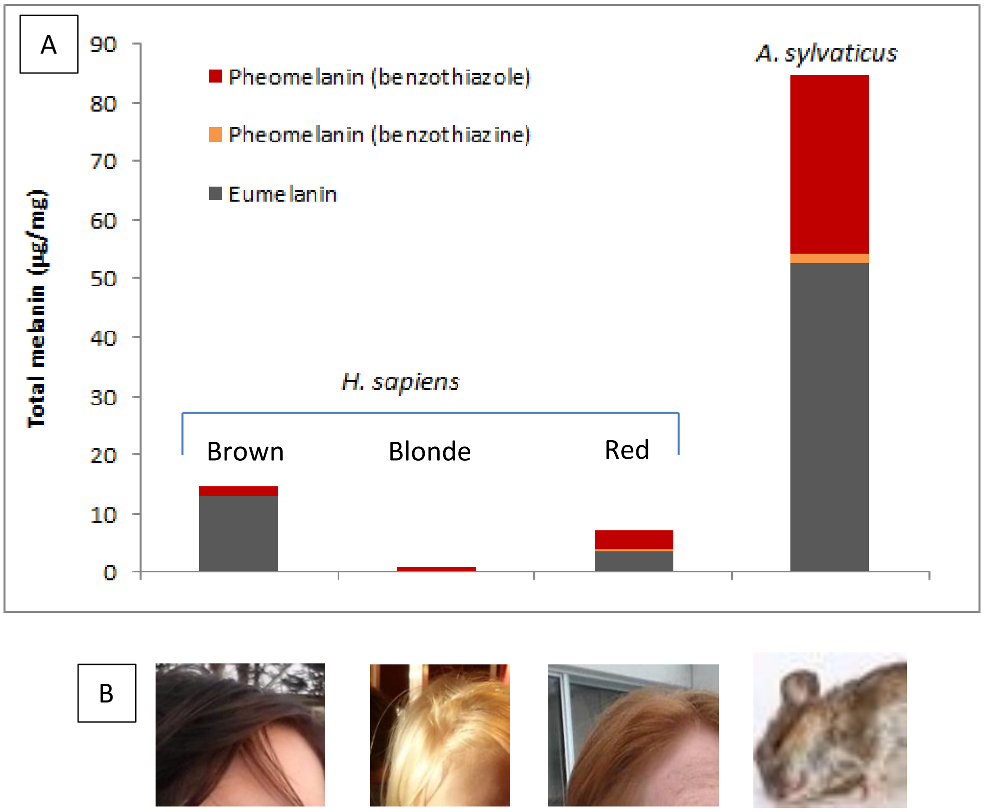

Supplementary Figure 6. **Melanin in extant tissue.** A) Melanin concentrations compared to B) specimen colour. (Photographs by R.A.W. [hair] and P.L.M. [fur])

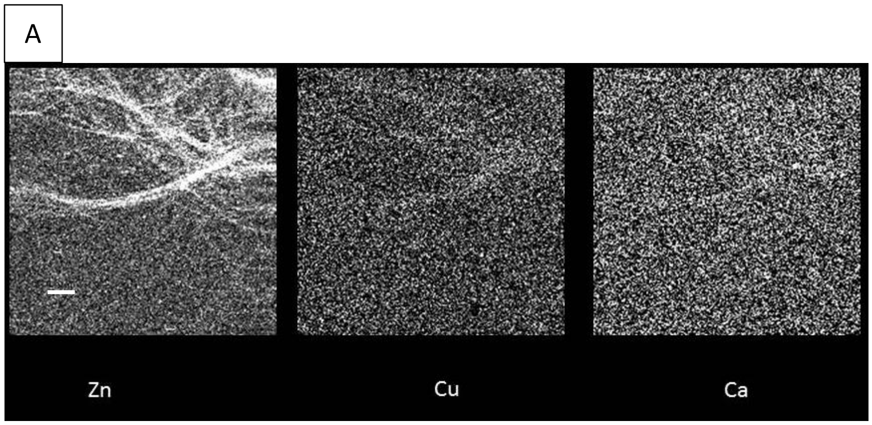

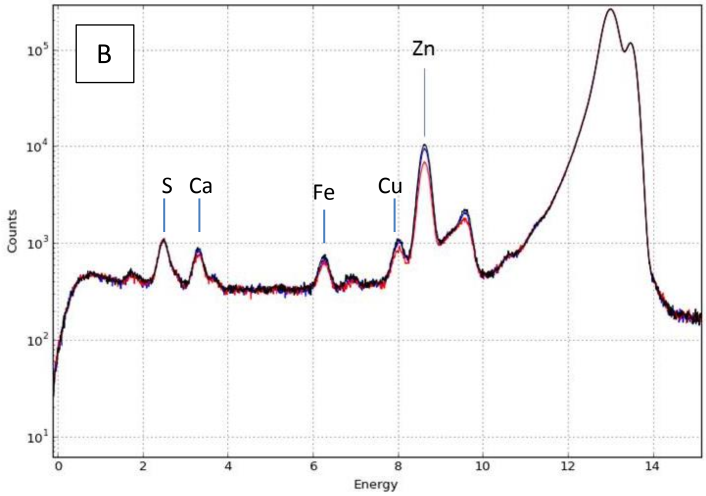

Supplementary Figure 7. **Red human hair analyses.** A) SRS-XRF map and B) example EDS spectrum of red human hair. Zn dominates the trace element loading. Map size 10 mm x 10 mm, scale bar = 1 mm.

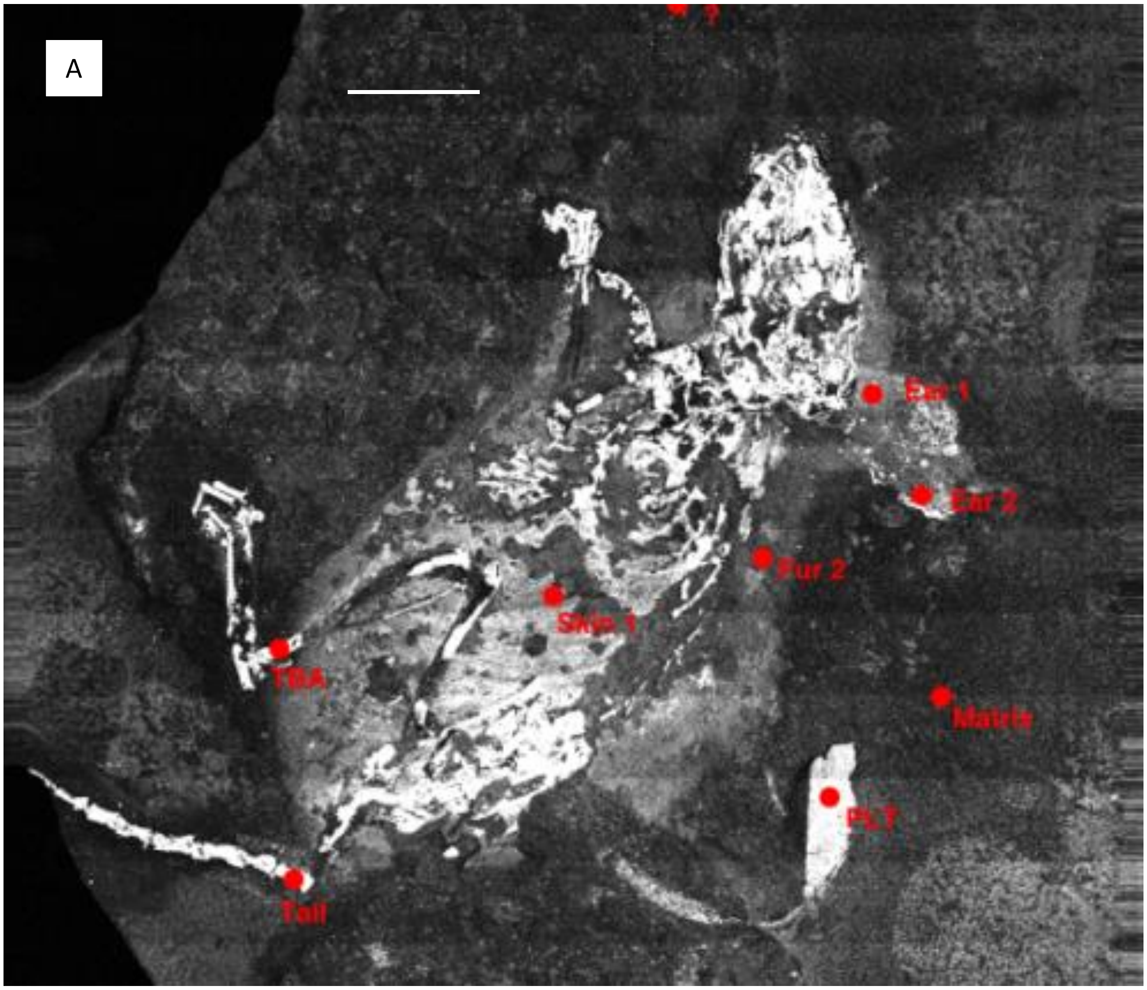

	Matrix_LZ	Skin1_LZ	Fur2_LZ	Tail_LZ	TBA_LZ	Ear1_LZ	Ear2_LZ	PLT_LZ
Al	2899	0	0	0	0	0	0	0
Si	21.54%	1.29%	6.86%	1.55%	2.55%	4095	3.57%	8.82%
P	0	3596	2721	37.30%	20.36%	6400	2.05%	3.56%
S	5.94%	7.75%	4%	3%	8%	6.78%	3.58%	6.52%
Cl	64.57	683.9	49.66	3532	5901	1526	289.8	349.5

Values in ppm except those marked with %.

Organic film estimated thickness = 24.5 microns

Supplementary Figure 8. **SRS-XRF point analyses.** A) Point locations for additional light element SRS-XRF point analyses of the lateral fossil indicated on the table. Errors on the tabled values are approximately 8% relative (2σ). A value of 0 indicates below the limit of detection (~40 ppm). Scale bar = 1 cm.

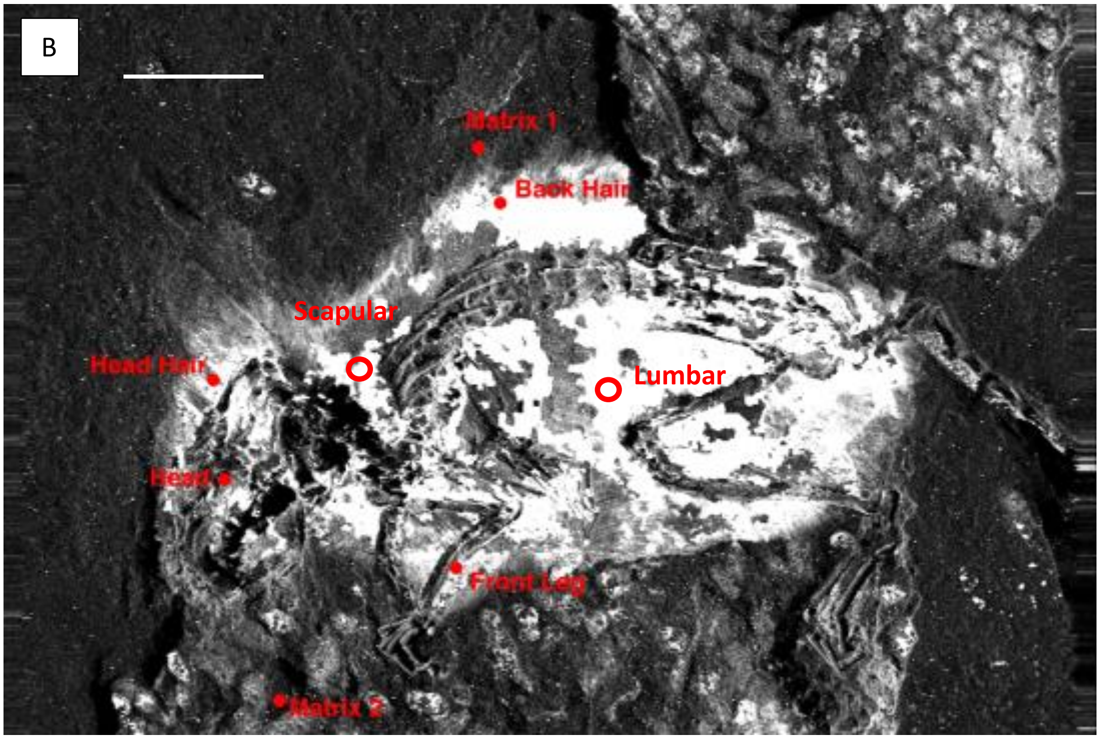

	Matrix 1	Matrix 2	Backhair		Headhair	
			L0	L1	L0	L1
Ca	8.06%	8.48%	0%	3.52%	0%	2.05%
Ti	123.6	315.2	2057	1496	1479	1056
V	0	0	67.58	37.42	1061	587.5
Cr	0	0	0	0	6.809	2.974
Mn	923.6	840.3	1535	537.1	699	244.7
Fe	2659	4450	0	2756	0	429.9
Ni	38.32	50.18	106.6	29.95	7.34	2.061
Cu	11.004	16.16	19.91	4.742	21.49	5.116
Zn	37.09	33.48	4110	835.8	850.2	172.9
Ga	8.259	6.67	317.1	55.75	43.71	7.686
Ge	3.359	2.602	157.3	24.19	18.14	2.789
As	4.309	9.175	106	14.38	22.5	3.053
Br	0.8264	2.082	57.58	6.287	12.66	1.382

Values in ppm except those marked with %.

Supplementary Figure 8 (continued). **SRS-XRF point analyses.** B) Point locations for heavy element SRS-XRF analyses (filled circles) and Zn K-edge EXAFS analyses (open circles) of the lateral fossil indicated on the zinc map, with concentration results table keyed to the map. L0 refers to the surface organic film, L1 refers to the underlying rock matrix. (Point analyses from SSRL, beamline 6-2; EXAFS from DLS, beamline I18.) Errors on the tabled values are approximately 4% relative (2σ) for most values, however as the values approach the limit of detection (~2 ppm) errors increase to 40% relative (2σ). A value of 0 indicates below the limit of detection. Scale bar = 1 cm.

C

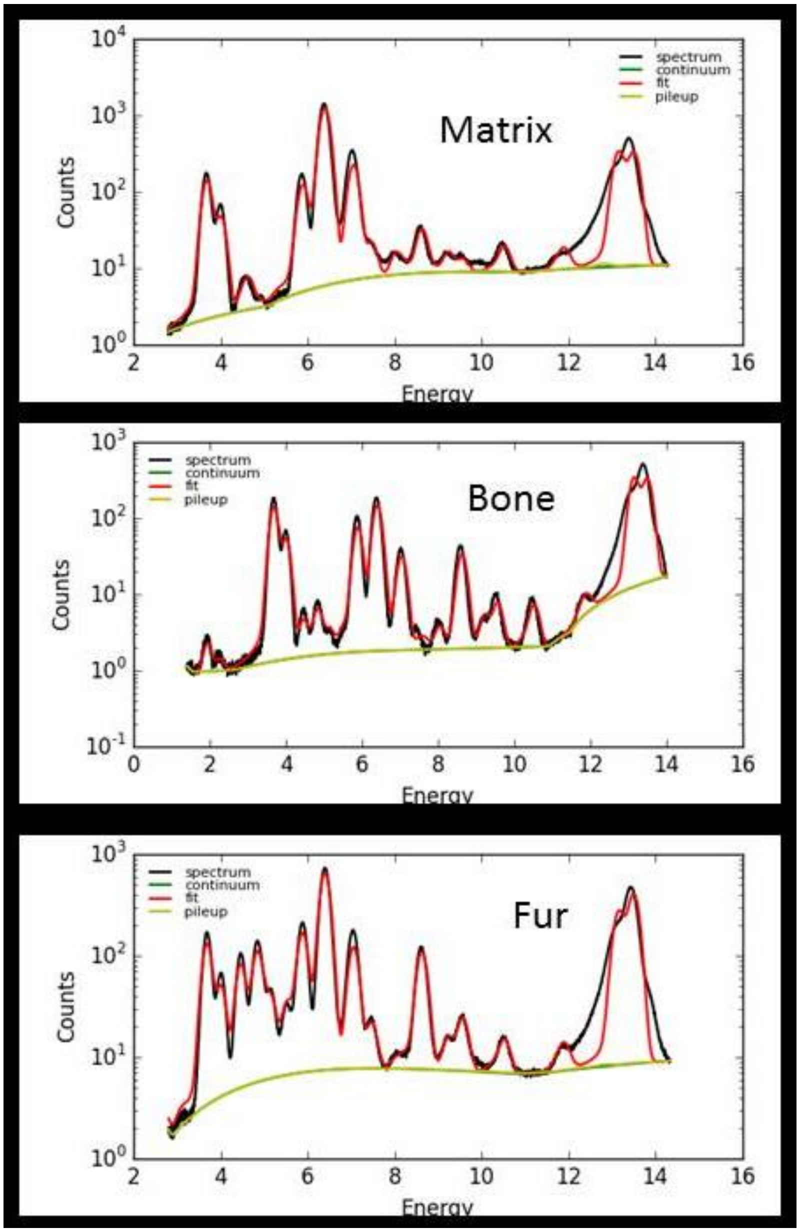

Supplementary Figure 8 (continued). **SRS-XRF point analyses.** C) Examples of SRS-XRF point analysis fits from the fossil material. The fossil fur is modelled as a two-layer analyte.

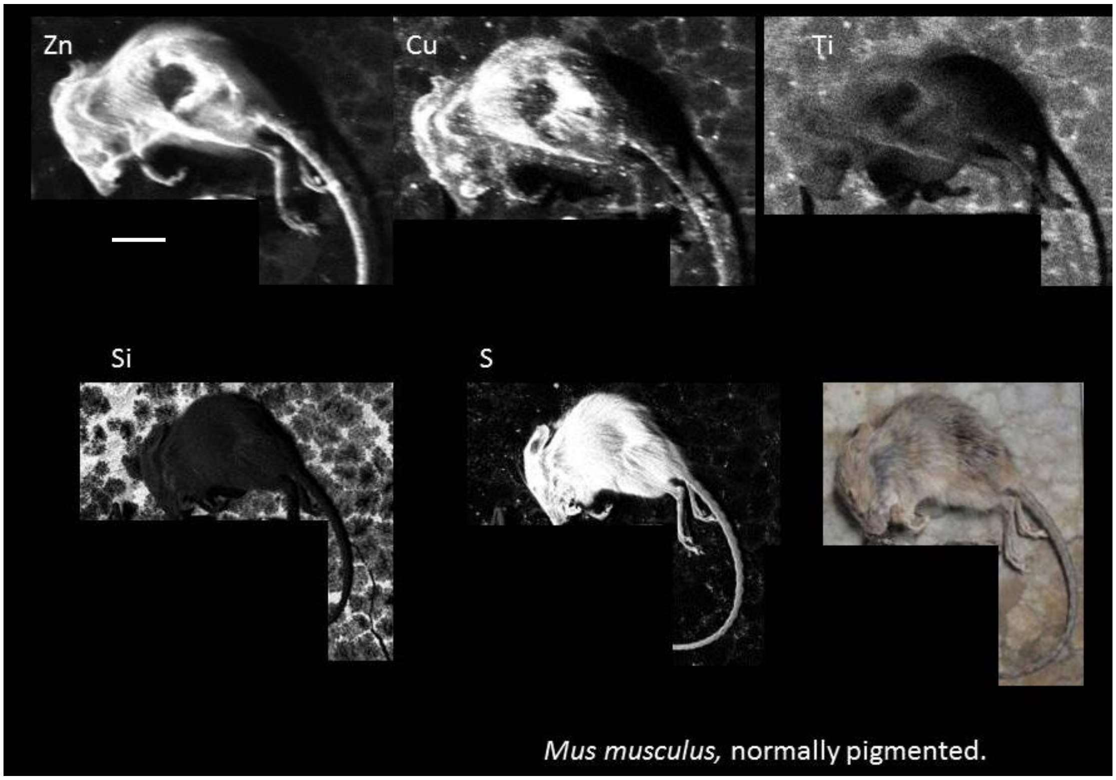

Supplementary Figure 9. **SRS-XRF map of normally pigmented *Mus musculus*.** Cu, Zn, and S are concentrated in the integument. Mosaic background is caused by a non-uniform composition mounting plate which is no longer in use. Scale bar = 1 cm. (Photograph by PLM.)

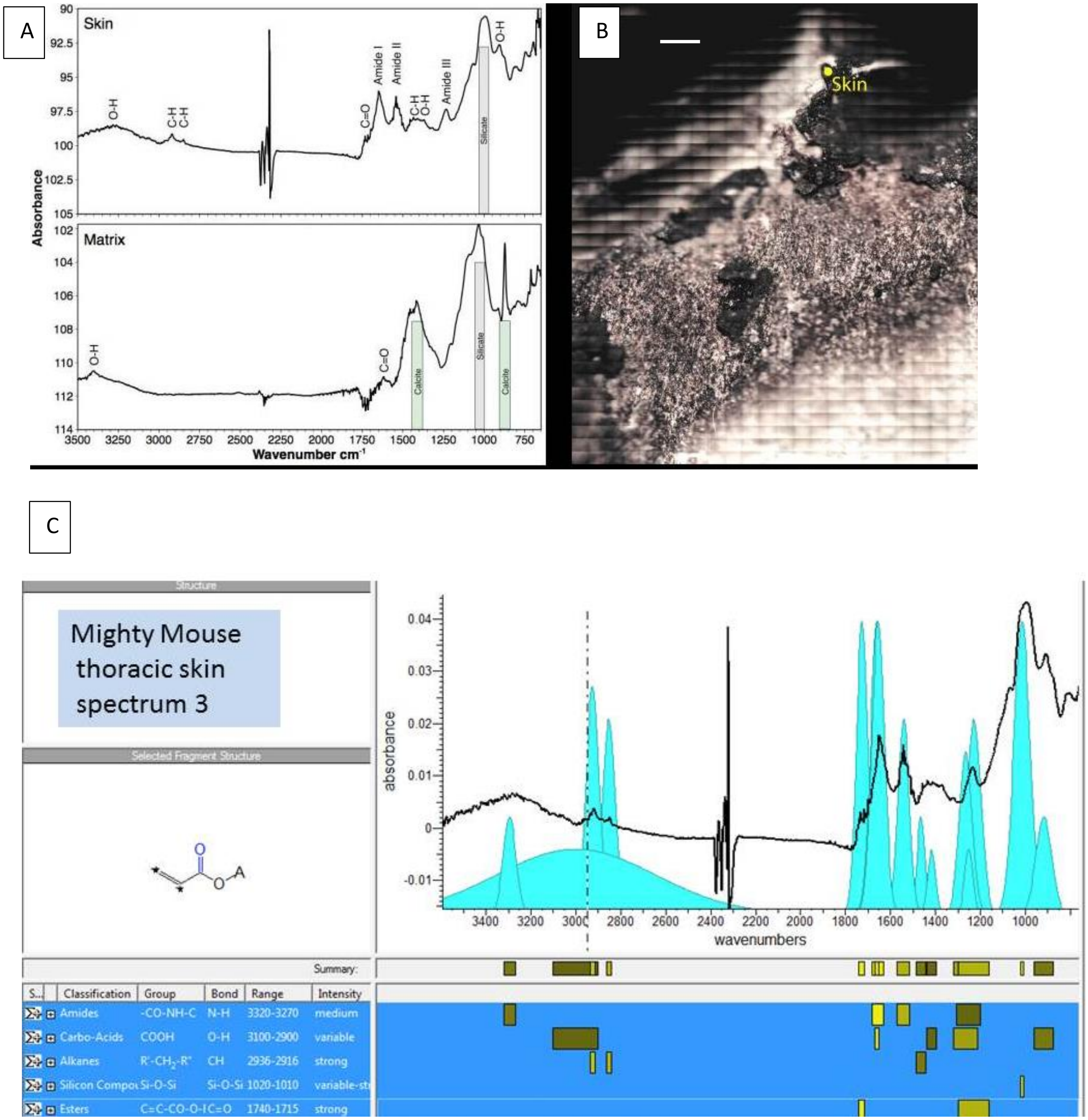

Supplementary Figure 10. **FTIR analyses.** A) Comparison of ATR-FTIR point analyses in the skin/fur area of the lateral fossil to the matrix. Peaks produced by inorganic silica and carbonate are labelled. B) Visible light photo mosaic of the skin/fur region analysed via FTIR with point location indicated. C) FTIR peak assignments for the organic functional groups in the fossil skin (Amides/Carboxylic acids/Alkanes/Silica Compounds/Esters). Scale bar = 1 mm.

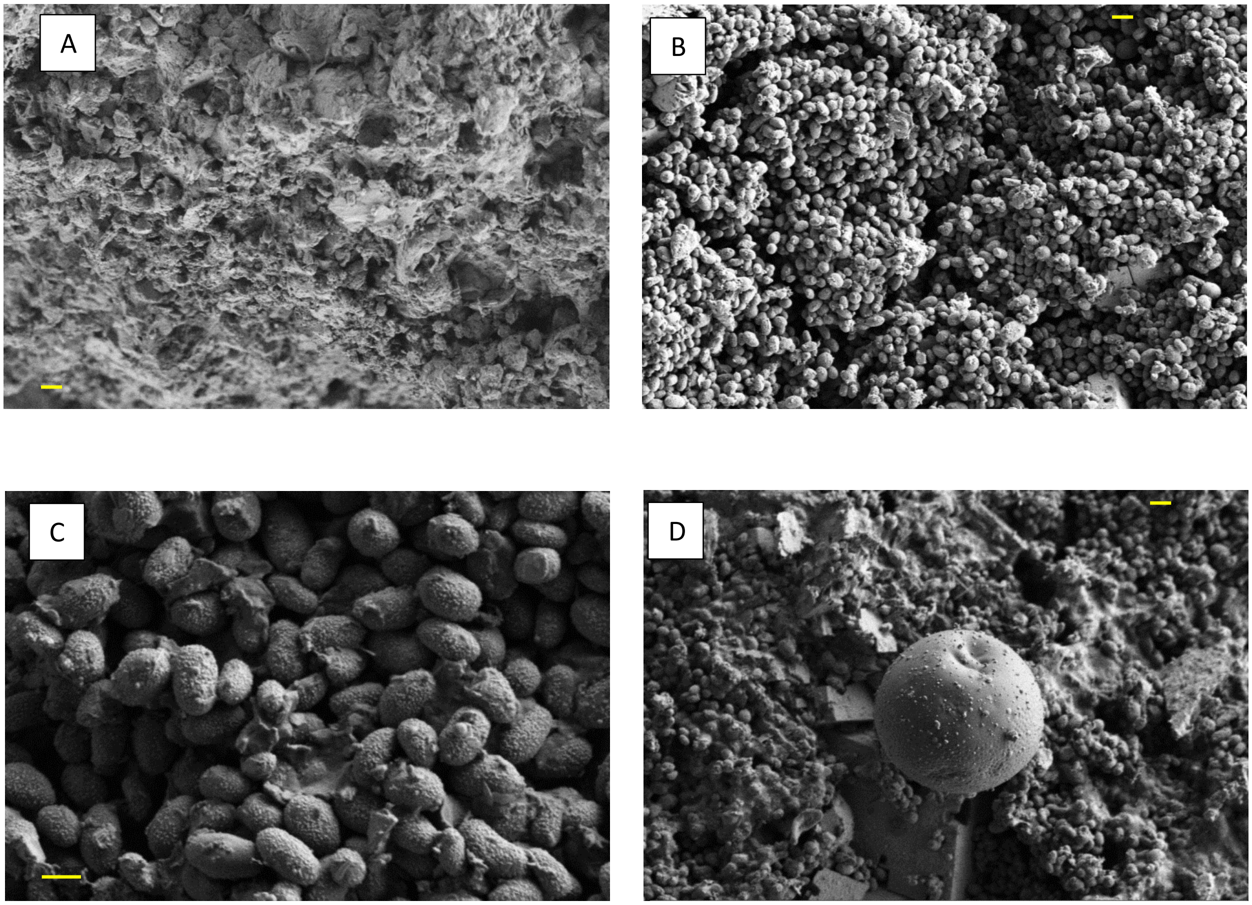

Supplementary Figure 11. **ESEM analyses.** A) ESEM image of the sedimentary matrix distal from the mouse fossil. B) ESEM image at same magnification from an area where the mouse fur would have been. Surface morphologies are completely different. C) Magnified ESEM image of possible melanosomal bodies which blanket the fossil fur areas. These microbodies are ubiquitous on the fossil integument. D) A large cyst on the fur. A,B,D; scale bar = 2 microns. C; scale bar = 1 micron.

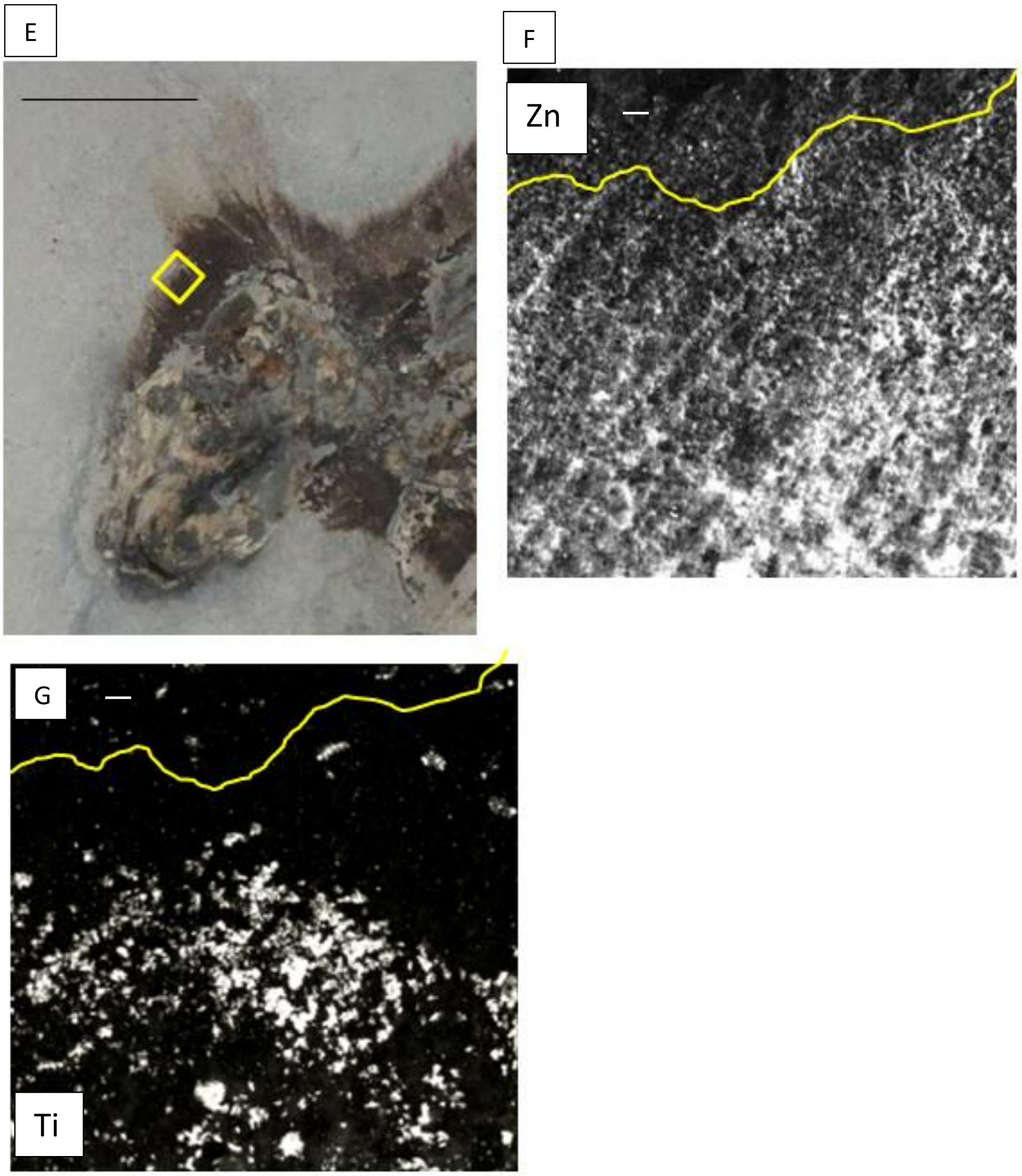

Supplementary Figure 11 (continued). **ESEM analyses.** E) Optical photographic detail of the lateral mouse skull (scale bar 1 cm). The area mapped using microfocus XRF is indicated by the yellow box. F) XRF microfocus map of Zn in the periphery of the integument. The yellow curve drawn in the Zn map delineates the transition between elevated Zn concentrations in the soft tissue and reduced Zn levels in the lithological matrix. Lineations running from lower left to upper right in this image are interpreted to be caused by the original texture of fur. (Microfocus scan dimension is 2 x 2 mm, pixel size is 2 x 5 μm, scale bar = 100 μm.) G) This XRF map of Ti is completely different from Zn and does not follow patterns expected for fur or skin, but rather is distributed as a diffuse set of discontinuous bright spots. (Scale bar = 100 μm.) These small discrete bodies are diatom tests and cysts. Ti is not typically important in the biochemistry of life, however unexplained high amounts of Ti have been reported from Willershausen in

the past[13] and this ESEM analysis reveals the cause. Diatoms are a unique group that biomineralize using Ti, because they can easily substitute Ti for Si in their tests and cysts. The Ti in the microfocus maps is indeed a part of a biological structure, but it results from Ti-rich tests and cysts adhering to the fur post-mortem as the animal fell through the water column and was covered with sediment at the lake bottom. This resolves the source of Ti within the Willershausen sediments and provides another example of how a combination of structural and chemical methods are required to make progress in unraveling complex bio-geochemical fossil specimens. (Photograph by P.L.M.)

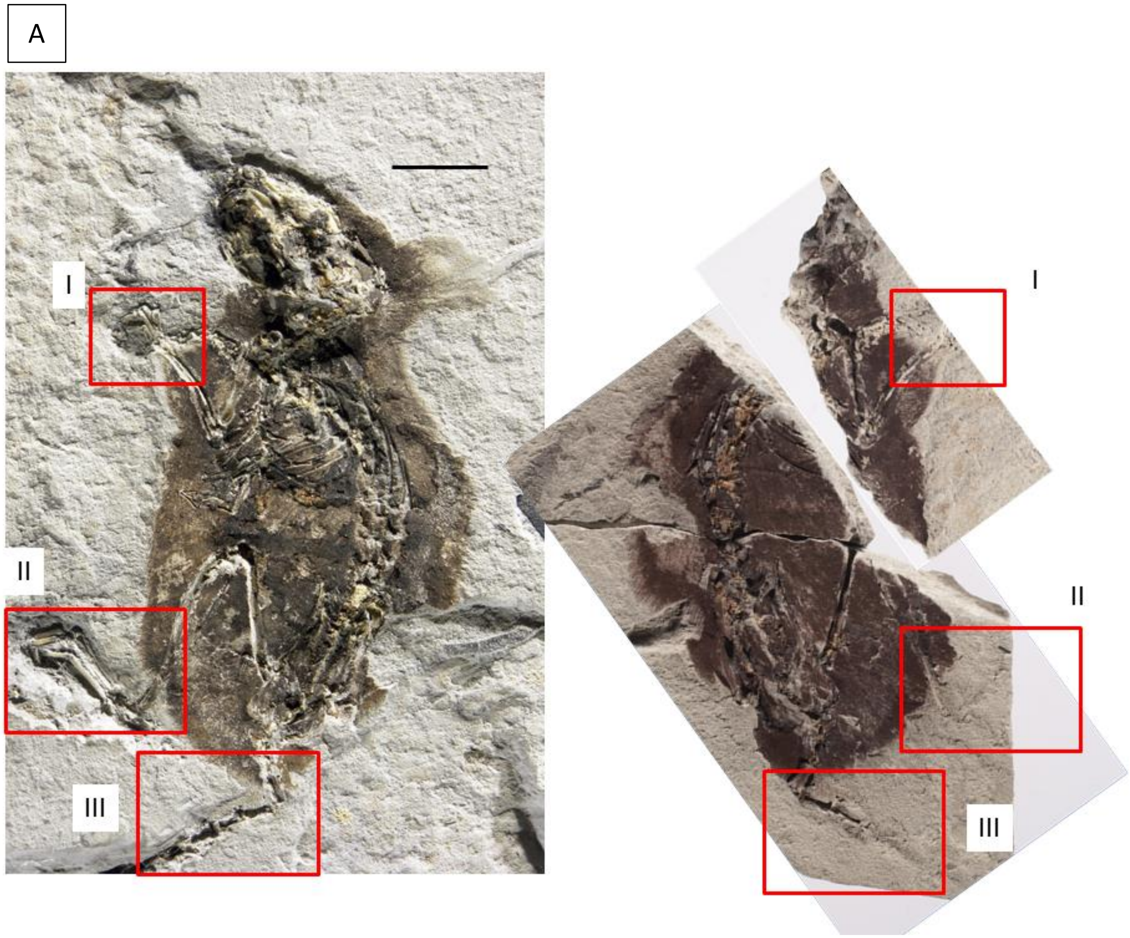

Supplementary Figure 12A. **Optical images to show details of fossil separation.** Optical photograph of the lateral fossil [GZG.W.20027b, left] compared to its counterparts [GZG.W.20027a(1), bottom right; GZG.W.20027a(2), top right]. Scale bar = 1 cm. Three regions are highlighted for comparison to check whether uneven separation of soft tissue between part and counterpart could explain the lack of chemical residue in the scanned images. (Copyright for these photos: GZG Museum / G. Hundertmark.)

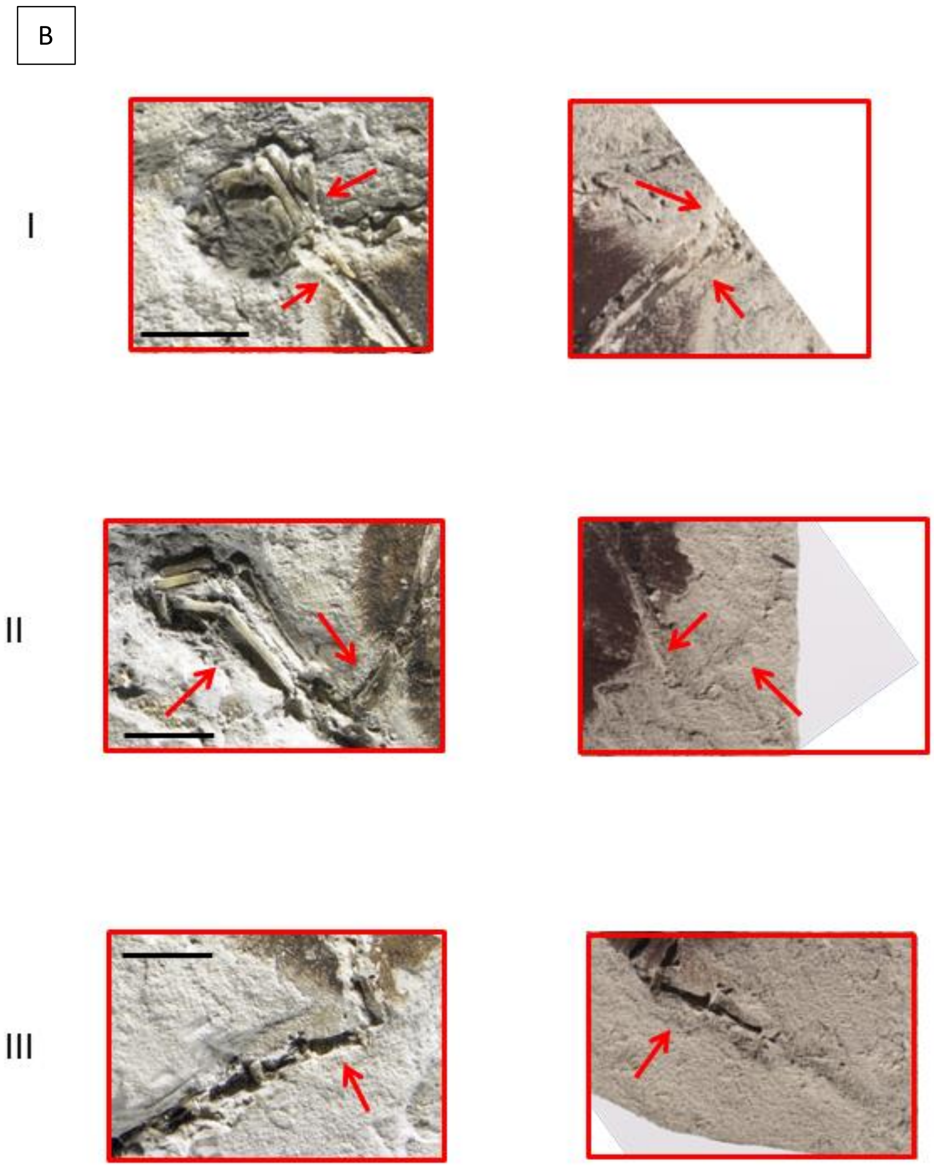

Supplementary Figure 12B (continued). **Optical images to show details of fossil separation.**Detailed photographic images of the scanned regions of the lateral fossil [GZG.W.20027b] presented on the left compared to its counterpart regions on the right [GZG.W.20027a(1), region I; GZG.W.20027a(2), regions II and III]. Scale bars = 0.5 cm. Arrows indicate expected locations of soft tissue. There is no evidence of uneven separation causing more soft tissue to be preserved on the counterpart in any of these three regions. Indeed, in all three cases more of the fossil residue appears on the left. Therefore the lack of pigment residue within these regions is most probably due to low levels in the original organism. (Copyright for these photos: GZG Museum / G. Hundertmark.)

C

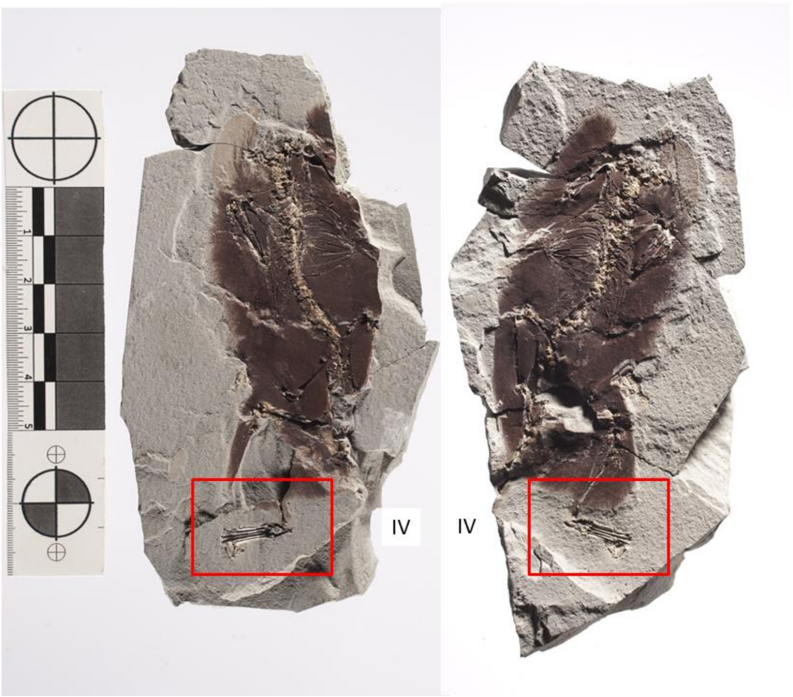

Supplementary Figure 12C (continued). **Optical images to show details of fossil separation.** Optical photograph of the dorsal fossil [GZG.W.17393a, left] compared to its counterpart [GZG.W.17393b, right]. Scale bar in cm. A fourth region is highlighted for comparison to check whether uneven separation of soft tissue between part and counterpart could explain the lack of chemical residue in the scanned images. (Copyright for these photos: GZG Museum / G. Hundertmark.)

D

IV

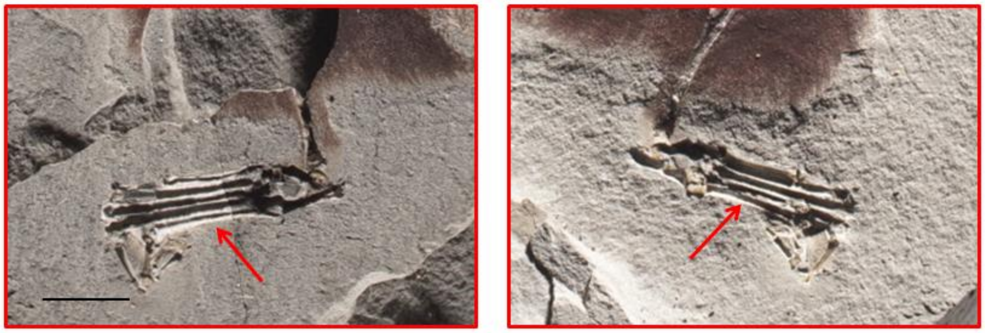

Supplementary Figure 12D (continued). **Optical images to show details of fossil separation.** Detailed photographic images of the scanned region IV of the dorsal fossil [GZG.W.17393a] presented on the left compared to its counterpart region on the right [GZG.W.17393b]. Scale bar = 0.5 cm. Arrows indicate expected locations of soft tissue. There is no evidence of uneven separation causing more soft tissue to be preserved on the counterpart. Therefore the lack of pigment residue within this region is also most probably due to low levels in the original organism. (Copyright for these photos: GZG Museum / G. Hundertmark.)

Supplementary Table 1. **Sulfur speciation and quantification.** Data are from normalized* XANES LCF analyses (2σ estimated errors on last digit given in brackets) and total sulfur XRF quantification (sulfur quantification 2σ error = ± 8% relative). All data from DLS I18.

Standards	***A. atavus*** **fossil 1**	***A. atavus*** **fossil 2**	**Graminoid plant fossil**	***A. atavus*** **bone**	**Sedimentary matrix**	***A. sylvaticus*** **red extant**	***M. musculus*** **albino extant**
Benzothiazole	**0.24 (3)**	**0.07 (4)**	0.00	0.03 (3)	0.00	**0.09 (2)**	0.06 (1)
Zn-cysteine	0.11 (8)	0.05 (4)	0.01 (1)	0.00	0.00		
Disulphide (ox_glu)	0.02 (2)	0.03 (3)	0.24 (6)	0.00	0.00	0.91 (2)	0.94 (1)
Meth. Sulfoxide	0.06 (2)	0.03 (3)	0.03 (3)	0.00	0.00		
Sulfate	0.57 (1)	0.82 (1)	0.71 (1)	0.97 (3)	1.0 (3)		
R-factor	0.013	0.013	0.015	0.096	0.090	0.018	0.010
Total S#	**5.52%**					**5.51%**	
dC/dt ($S_{R\text{->}O}$)*	~8 x 10^{-21}						
t_f	66 m.y.						
dC/dt (quartz)*	~2 x 10^{-14}						
#wt. percent							
*mol g^{-1} s^{-1}							

*LCF normalization was accomplished by setting the edge jump between the pre-edge and post edge to be equal to 1. Pre-edge was typically -20 to -10 eV relative to the critical energy. The post-edge normalization region varied depending on the analyte. Typically the post-edge was taken as 8 to 30 eV above the critical energy, extending between 15 to 150 eV above the critical energy. Beam induced photo-oxidation is a problem with sulfur speciation analysis using a synchrotron beam, and we performed many preliminary scans in order to optimize scan times and scan ranges such that the XANES spectra were not affected by oxidation but were wide enough in energy to allow normalization above the edge for all of the specimens analyzed.

Supplementary Table 2. **Calibrated energies for dominant sulfur absorption peaks**. Data are for standards, with schematic diagrams of the important sulfur species at right.

Assigned S K-edge XANES peaks for standards		
Cystine	2471.42	**S-S**
Oxidized glutathione	2471.51	**S-S**
Cu-cysteine	2471.64	S-Cu
Zn-cysteine	2471.65	S-Zn
Cysteine	2471.77	S-H/S-C
Methionine	2472.20	S-C
Reduced glutathione	2472.21	S-H/S-C
Sphalerite	2472.41	S-Zn
Meth. Sulfoxide	2472.55	S-C
Cu-cysteine	2473.23	S-C
Oxidized glutathione	2473.34	S-C
Cystine	2473.38	S-C
Zn-cysteine	2473.47	S-C
Meth. Sulfoxide	2475.15	S=O
Sulfone	2478.26	2S=O/2S-R
Taurine	2479.48	S-O/2S=O/S-C
Sulfate	2481.19	4 S=O

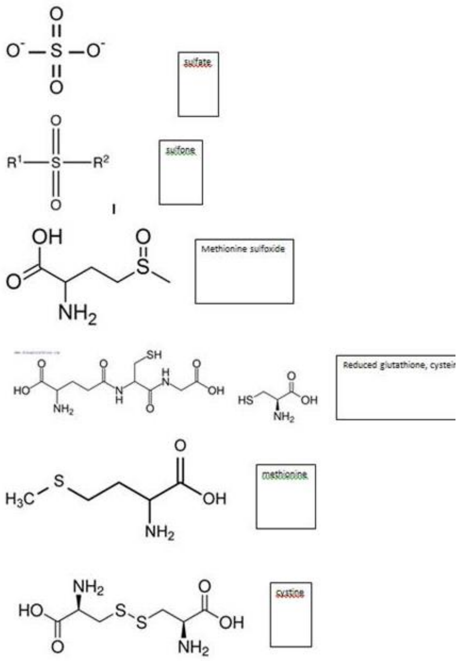

Supplementary Table 3. **ESEM EDS analyses of *A. apodemus* lateral fossil.** Data are semi-quantitative standardless results, with 2σ errors approximately 10% relative.

	Mouse EDS analyses		
	Element wt. %		
	Ear	Fur	Matrix
C	**13.67**	**36.91**	6.11
O	48.38	37.29	44.82
Na	0.45	1	0.28
Mg	11.31	1.2	4.63
Al	0.67	0.64	3.45
Si	0.99	1.01	5.6
P	0.4	**2.23**	0.42
S	1.14	**7.04**	1.24
K	0.23	0.34	0.88
Ca	22.19	9.46	30.29
Mn	0.58	0.29	0.53
Fe		0.62	1.73
Cl		0.32	
Ti		**1.64**	←

Supplementary references

1. Sánchez-Ferrer, A., Rodríguez-López, J.N., García-Cánovas, F., & García-Carmona, F.. Tyrosinase: a comprehensive review of its mechanism. *Biochim Biophys Acta* **1247**, 1–11 (1995).
2. Trinkaus J.P. Factors concerned in the response of melanoblasts to estrogen in the Brown Leghorn fowl. *J. Exptl. Zool*. **109**, 135-170 (1948).
3. Ito, S. A chemist's view of melanogenesis. *Pigment Cell Research* **16**, 230-236 (2003).
4. Land, E.J., Ito, S., Wakamatsu, K., & Riley, P.A. Rate Constants for the First Two Chemical Steps of Eumelanogenesis, *Pigment Cell Research* **16**, 487-493 (2003).
5. Simon, J.D., Peles, D., Wakamatsu, K., & Ito, S. Current challenges in understanding melanogenesis: bridging chemistry, biological control, morphology, and function. *Pigment Cell & Melanoma Research* **22**, 563–579 (2009).
6. Palumbo, A., d'Ischia, M., Misuraca, G., Prota, G., & Schultz, T.M. Structural modifications in biosynthetic melanins induced by metal ions. *Biochemica et Biophysica Acta (BBA)- General Subjects* **694**(2), 193-199 (1988).
7. Riley, P.A. Melanin. *Int. J. Biochem. Cell Biol.* **29**, 1235–1239 (1997).
8. Willier, B.H., & Rawles, M.E. The Control of Feather Color Pattern by Melanophores Grafted from One Embryo to Another of a Different Breed of Fowl. *Physiological Zoology* **13**, no. 2, 177-201 (1940)
9. Ralph, C.L. The control of color in birds. *American Zoologist* **9**, 521-530 (1969).
10. Jimbow, K., Quevedo, W.C., Fitzpatrick, T.B., & Szabo, G. Some aspects on melanin biology: 1950-1975. *Journal of investigative dermatology* **67**(1), 72-89 (1976).
11. Meischner, D., & Paul, J. Willershausen disused clay pit–reconstruction of a meromictic Pliocene pond environment from its sediments and fossils. In Field Guide Harz Mountains. *International Symposium on Environmental Biogeochemistry, Wolfenbüttel*, pp. 6-12, (1977).
12. Rietschel, S., & Storch, G. Aussergewöhnlich erhaltene Waldmäuse (Apodemus atavus Heller, 1936) aus dem Ober-Pliozän von Willershausen am Harz. *Senckenbergiana lethaea*, *54*(5/6), 491-519 (1974).
13. Meischner, D., & Paul, J. *Die pliozane Fossilfundstatte Naturdenkmal Tongrube Willershausen: Courier Forschungs-Institut Senckenberg* **56**, 147-152 (1982).
14. Meischner, D. Die ehemalige Tongrube Willershausen, ein Naturdenkmal von weltweiter Bedeutung. In: Jaeckel, H., Diesner, R., Hillebrecht, W. (Eds.), *Willershausen am Harz — Umrisse einer Dorfgeschichte,* pp. 9–30 (1994).
15. Meischner, D. Klassische Aufschlusse im Tertiar Siid-Nied-ersachsens, Lokalitat 2: Willershausen. 65. Jahrestagung der Palaontologischen Gesellschaft, Hildesheim, 25-30 September 1995, Exkursionsfiihrer. *Terra Nostra* **5**, 217-228 (1995).
16. Briggs, D.E.G., Stankiewicz, A.B., & Meischner, D. Taphonomy of arthropod cuticles from Pliocene Lake Sediments, Willerhausen, Germany. *Palaios* **13**(4), 386-394 (1998).
17. Krasske, G. Diatomeen aus dem Oberpliocan von Willershausen (Biologie eines jungtertiaren Teiches in Sudhannover II.Teil. *Arch. Hydrobiol*. **24**, 431–447 (1932).
18. Ferguson, D.K., & Knobloch, E.A. A fresh look at the rich assemblage from the Pliocene sink-hole of Willershausen, Germany. *Review of Palaeobotany and Palynology* **101**, 271-286 (1998).
19. Flannery, M.B., Stott, A.W., Briggs, D.E.G., & Evershed, R.P. Chitin in the fossil record: identification and quantification of D-glucosamine. *Organic Geochemistry* **32**(5), 745-754 (2001).
20. Kordos, L. "Das Untere Miozän von Ipolytarnóc in Ungarn." *Europäische Fossillagerstätten*. Springer Berlin Heidelberg. 184-213 (2000).
21. Chinga, G., & Syverud, K. Quantification of paper mass distributions within local picking areas. *Nordic Pulp and Paper Res. J.* **22**, 441 (2007).
